# Survey of severe acute respiratory syndrome coronavirus 2 in captive and free-ranging wildlife from Spain

**DOI:** 10.1186/s13567-024-01348-0

**Published:** 2024-07-19

**Authors:** Leira Fernández-Bastit, David Cano-Terriza, Javier Caballero-Gómez, Adrián Beato-Benítez, Antonio Fernández, Daniel García-Párraga, Mariano Domingo, Cecilia Sierra, Rocío Canales, Santiago Borragan, Manuel de la Riva-Fraga, Rafael Molina-López, Óscar Cabezón, Maria Puig-Ribas, Johan Espunyes, Daniel B. Vázquez-Calero, Júlia Vergara-Alert, Ignacio García-Bocanegra, Joaquim Segalés

**Affiliations:** 1 Unitat Mixta d’Investigació IRTA-UAB en Sanitat Animal. Centre de Recerca en Sanitat Animal (CReSA), Campus de la Universitat Autònoma de Barcelona (UAB), 08139 Bellaterra, Barcelona Spain; 2 IRTA, Programa de Sanitat Animal, Centre de Recerca en Sanitat Animal (CReSA), Campus de la Universitat Autònoma de Barcelona (UAB), 08193 Bellaterra, Barcelona Spain; 3 Departamento de Sanidad Animal, Grupo de Investigación en Sanidad Animal y Zoonosis (GISAZ), UIC Zoonosis y Enfermedades Emergentes ENZOEM, Universidad de Córdoba, 14014 Córdoba, Spain; 4 CIBERINFEC, ISCIII-CIBER de Enfermedades Infecciosas, Instituto de Salud Carlos III, 28029 Madrid, Spain; 5 Maimonides Institute for Biomedical Research of Cordoba, Reina Sofía University Hospital, University of Córdoba, 14004 Córdoba, Spain; 6 Atlantic Cetacean Research Center, Institute of Animal Health, University of Las Palmas de Gran Canaria, 35001 Las Palmas, Trasmontaña Spain; 7 Research Department, Fundación Oceanografic de la Comunitat Valenciana, Ciudad de las Artes y las Ciencias, 46013 Valencia, Spain; 8 Veterinary Pathology Diagnostic Service, Autonomous University of Barcelona, 08193 Bellaterra, Barcelona Spain; 9 Departament de Sanitat I Anatomia Animals, Facultat de Veterinària, Universitat Autònoma de Barcelona, 08193 Bellaterra, Barcelona Spain; 10 Selwo Aventura, 29680 Estepona, Málaga Spain; 11 Selwo Marina, 29630 Benalmádena, Málaga Spain; 12 Mundomar Benidorm, 03503 Benidorm, Alicante Spain; 13 Parque de la Naturaleza de Cabárceno, 39690 Obregón, Cantabria Spain; 14 Faunia, 28032 Madrid, Spain; 15 Centre de Fauna de Torreferrussa, Àrea de Gestió Ambiental Servei de Fauna I Flora, Forestal Catalana, 08130 Santa Perpètua de Mogoda, Barcelona Spain; 16 Wildlife Conservation Medicine Research Group (WildCoM), Departament de Medicina I Cirugia Animals, Universitat Autònoma de Barcelona, 08193 Bellaterra, Barcelona Spain; 17 Veterinary Clinic of Exotic Pets ARACAVIA, 29007 Málaga, Spain

**Keywords:** SARS-CoV-2, wildlife, surveillance, cetacean, bottlenose dolphin, zoonosis

## Abstract

Severe acute respiratory syndrome coronavirus 2 (SARS-CoV-2), considered a zoonotic agent of wildlife origin, can infect various animal species, including wildlife in free-range and captive environments. Detecting susceptible species and potential reservoirs is crucial for preventing the transmission, spread, genetic evolution, and further emergence of viral variants that are major threats to global health. This study aimed to detect exposure or acute infection by SARS-CoV-2 in 420 animals from 40 different wildlife species, including terrestrial and aquatic mammals, from different regions of Spain during the 2020–2023 coronavirus disease 19 (COVID-19) pandemic. In total, 8/137 animals were positive for SARS-CoV-2 antibodies against the receptor binding domain and/or viral nucleoprotein according to independent ELISAs. However, only one ELISA-positive sample of a captive bottlenose dolphin (*Tursiops truncatus*) tested positive for SARS-CoV-2 neutralizing antibodies with a low titre (SNT_50_ 38.15) according to a virus neutralization test. Cetaceans are expected to have a high risk of infection with SARS-CoV-2 according to early predictive studies due to the similarity of their angiotensin converting enzyme 2 cell receptor to that of humans. Moreover, of 283 animals analysed for SARS-CoV-2 RNA using RT-qPCR, none tested positive. Our results reinforce the importance of considering cetaceans at risk for SARS-CoV-2 infection and support taking preventive biosecurity measures when interacting with them, especially in the presence of individuals with suspected or confirmed COVID-19. Although most animals in this study tested negative for acute infection or viral exposure, ongoing surveillance of wildlife species and potentially susceptible animals is important to prevent future spillover events and detect potential novel reservoirs.

**Supplementary Information:**

The online version contains supplementary material available at 10.1186/s13567-024-01348-0.

## Introduction

Wildlife has been proposed as the source of significant emerging viral diseases in humans (zoonosis), including coronavirus disease 19 (COVID-19), which is caused by severe acute respiratory syndrome coronavirus 2 (SARS-CoV-2) [1]. The emergence of these diseases may, in part, be attributed to human behaviour (e.g., hunting practices or consumption of wild meat), population growth and urbanization, and the modification of the wildlife habitat structure, which leads to evident human-animal interactions [2]. Although humans are the main hosts of SARS-CoV-2, from the outset of COVID-19, SARS-CoV-2 has demonstrated the ability to cross species barriers in free-ranging and captive scenarios [3].

According to early in silico studies, nonhuman primates (NHPs), several carnivore species (mainly felines), and cetaceans are considered at moderate or high risk of infection with SARS-CoV-2 [4–6]. These studies were mainly based on comparative and structural analyses of the sequence of angiotensin-converting enzyme 2 (ACE2), the host cell receptor of SARS-CoV-2 [7]. The ACE2 sequence of NHP, carnivores and cetaceans has been demonstrated to have high homology with human ACE2 (hACE2), which is also considered a critical amino acid residue for binding with the SARS-CoV-2 receptor binding domain (RBD) [4, 6, 8]. Additionally, predictive results for the risk of infection based on comparative analysis of the transmembrane serine protease 2 (TMPRSS2) sequence of these species were consistent with those obtained from the ACE2 sequence [6]. TMPRSS2 is a protease that facilitates membrane fusion of cell membranes by S protein priming and subsequent viral entry [7]. To date, natural and experimental SARS-CoV-2 infections have confirmed the ability of the virus to infect many NHP and carnivore species [3, 9].

Zoological parks are scenarios in which SARS-CoV-2 animal infections have been documented globally during the pandemic. Most infections were described in great apes (*Gorilla gorilla*), tigers (*Panthera tigris*), lions (*Panthera leo*), and a variety of large and medium-sized felines [3]. Other mammals, mainly carnivores, have also been infected under captive conditions worldwide, including species from the families *Atelidae* (brown-headed spider monkey [*Ateles fusciceps*]), *Canidae* (red fox [*Vulpes vulpes*])*, Hyaenidae* (spotted hyena [*Crocuta crocuta*]), *Hippopotamidae* (hippopotamus [*Hippopotamus amphibius*])*, Mustelidae* (American mink [*Neogale vison*]; Asian small-clawed otter [*Aonyx cinereus*])*, Rhinocerotidae* (white rhinoceros [*Ceratotherium simum*]*)*, and *Viverridae* (South American coati [*Nasua nasua*]) [10–12]. Sequencing analysis and/or epidemiological history supported reverse zoonosis (human-to-animal transmission) as the origin of these animal infections [3].

Although animals living in a free-range environment are rarely as close to humans as domesticated or captive animals are, the risk of SARS-CoV-2 infection in wildlife has also been proven [6, 13, 14]. Human household waste, SARS-CoV-2-contaminated elements (e.g., food and water), and contact with other susceptible animals (e.g., farmed minks) are potential sources of infection in free-ranging wild animals [15–17]. In this regard, many spillover events from humans to white-tailed deer (WTD; *Odocoileus virginanus*) and even from the WTD back to humans have been described in the United States and Canada based on sequencing analysis [18–20]. WTD are highly abundant in urban and peri-urban areas in North America and are in close contact with humans and human-produced waste. Of concern, SARS-CoV-2 and its variants can infect, persist, adapt and be transmitted within the WTD population, suggesting that this species could serve as a reservoir for SARS-CoV-2 [18, 19, 21]. Mustelid species have also been exposed and/or infected by SARS-CoV-2 in the wild [15, 17, 22]. *Mustelidae* species have been involved in one of the most important SARS-CoV-2 animal events to date owing to the number of outbreaks on mink farms in multiple countries (the Netherlands, Denmark, the United States, Canada, France, Greece, Italy, Spain, Sweden, Poland and Lithuania) [3]. Farming has been shown to favour the spread of SARS-CoV-2 in other species, as indicated by the recently reported outbreak of the SARS-CoV-2 Delta (B.1.617.2) variant in farmed beavers (Order Rodentia; *Castor fiber*) in Mongolia [23]. In this sense, several rodent species have shown susceptibility to a variety of SARS-CoV-2 variants (Alpha [B.1.1.7], Beta [B.1.351], Gamma [P.1] and Omicron [B.1.1.529]), although not to the ancestral SARS-CoV-2 lineage (B.1.) [24–26]. In Northern Spain, the populations of two wild species of farming origin, the American mink and the coypu (order Rodentia, *Myocastor coypus*), have significantly increased in recent years and are considered exotic invasive species. Both species live near aquatic environments, representing a threat to autochthonous biodiversity [27–29]. However, SARS-CoV-2 exposure and infection have yet to be evaluated for most exotic and native wild species in Spain.

The present work aimed to investigate SARS-CoV-2 exposure and infection of different free-ranging and captive wildlife species during the COVID-19 pandemic (from 2020 to 2023) in Spain.

## Materials and methods

### Animals and samples

A total of 420 animals (119 captive and 301 free-ranging) from 40 different species were opportunistically sampled during the 2020–2023 COVID-19 pandemic to detect SARS-CoV-2 RNA or specific antibodies (Table 1).

**Table 1:** **Wild Animals tested for SARS-CoV-2 RNA and/or antibodies**

Animal species	Family	Sample size	Animal source	SARS-CoV-2 RNA detection	SARS-CoV-2 antibody detection
Red panda (*Ailurus fulgens*)	Ailuridae	3	Captive zoo	NA	3
Fennec fox (*Vulpes zerda*)	Canidae	2	Captive zoo	NA	2
Grey wolf (*Canis lupus*)	Canidae	1	Captive zoo	NA	1
Iberian wolf (*Canis lupus signatus*)	Canidae	1	Captive zoo	NA	1
Red fox (*Vulpes vulpes*)	Canidae	15	Free-ranging	15	NA
Atlantic spotted dolphin (*Stenella frontalis*)	Delphinidae	1	Free-ranging	NA	1
Bottlenose dolphin (*Tursiops truncatus*)	Delphinidae	46	Captive zoo	NA	48
		2	Free-ranging		
Killer whale (*Orcinus orca*)	Delphinidae	8	Captive zoo	NA	8
Risso’s dolphin (*Grampus griseus*)	Delphinidae	1	Free-ranging	NA	1
Striped dolphin (*Stenella coeruleoalba*)	Delphinidae	14	Free-ranging	NA	14
African lion (*Panthera leo*)	Felidae	7	Captive zoo	NA	7
Asian tiger (*Panthera tigris tigris*)	Felidae	1	Captive zoo	NA	1
Asiatic lion (*Panthera leo persica*)	Felidae	2	Captive zoo	NA	2
Chetaah (*Acinonyx jubatus*)	Felidae	4	Captive zoo	NA	4
European wildcat (*Felis silvestris silvestris*)	Felidae	1	Free-ranging	1	NA
Jaguar (*Panthera onca*)	Felidae	3	Captive zoo	NA	3
Ocelot (*Leopardus pardalis*)	Felidae	1	Captive zoo	NA	1
Persian leopard (*Panthera pardus saxicolor*)	Felidae	2	Captive zoo	NA	2
Sri Lankan leopard (*Panthera pardus kotiya*)	Felidae	2	Captive zoo	NA	2
Sumatran tiger (*Panthera tigris sumatrae*)	Felidae	2	Captive zoo	NA	2
Spotted hyena (*Crocuta crocuta*)	Hyaenidae	2	Captive zoo	NA	2
Striped skunk (*Mephitis mephitis*)	Mephitidae	2	Captive zoo	NA	2
Beluga whale (*Delphinapterus leucas*)	Monodontidae	1	Captive zoo	NA	1
American mink (*Neogale vison*)	Mustelidae	141	Free-ranging	141	NA
Asian small-clawed otter (*Aonyx cinereus*)	Mustelidae	1	Captive zoo	NA	1
Beech marten (*Martes foina*)	Mustelidae	2	Free-ranging	2	NA
Eurasian badger (*Meles meles*)	Mustelidae	48	Free-ranging	48	NA
Eurasian otter (*Lutra lutra*)	Mustelidae	25	Free-ranging	25	NA
Coypu (*Myocastor coypus*)	Myocastoridae	48	Free-ranging	48	NA
California sea lion (*Zalophus californianus*)	Otariidae	4	Captive zoo	NA	4
South American sea lion (*Otaria flavescens*)	Otariidae	9	Captive zoo	NA	9
Grey seal (*Halichoerus grypus*)	Phocidae	1	Captive zoo	NA	1
Harbor seal (*Phoca vitulina*)	Phocidae	1	Captive zoo	NA	1
Asian black bear (*Ursus thibetanus*)	Ursidae	1	Captive zoo	NA	1
Black bear (*Ursus americanus*)	Ursidae	1	Captive zoo	NA	1
Brown bear (*Ursus arctos*)	Ursidae	7	Captive zoo	NA	7
Giant panda (*Ailuropoda melanoleuca*)	Ursidae	1	Captive zoo	NA	1
Sun bear (*Helarctos malayanus*)	Ursidae	1	Captive zoo	NA	1
Binturong (*Arctictis binturong*)	Viverridae	1	Captive zoo	NA	1
Common genet (*Genetta genetta*)	Viverridae	1	Captive zoo	NA	1
		3	Free-ranging	3	NA
Total		420		283	137

The sample size, animal source (captive or free-ranging) and animals tested for the detection of SARS-CoV-2 RNA or antibodies are indicated.

NA: not available.

**Figure 1:**
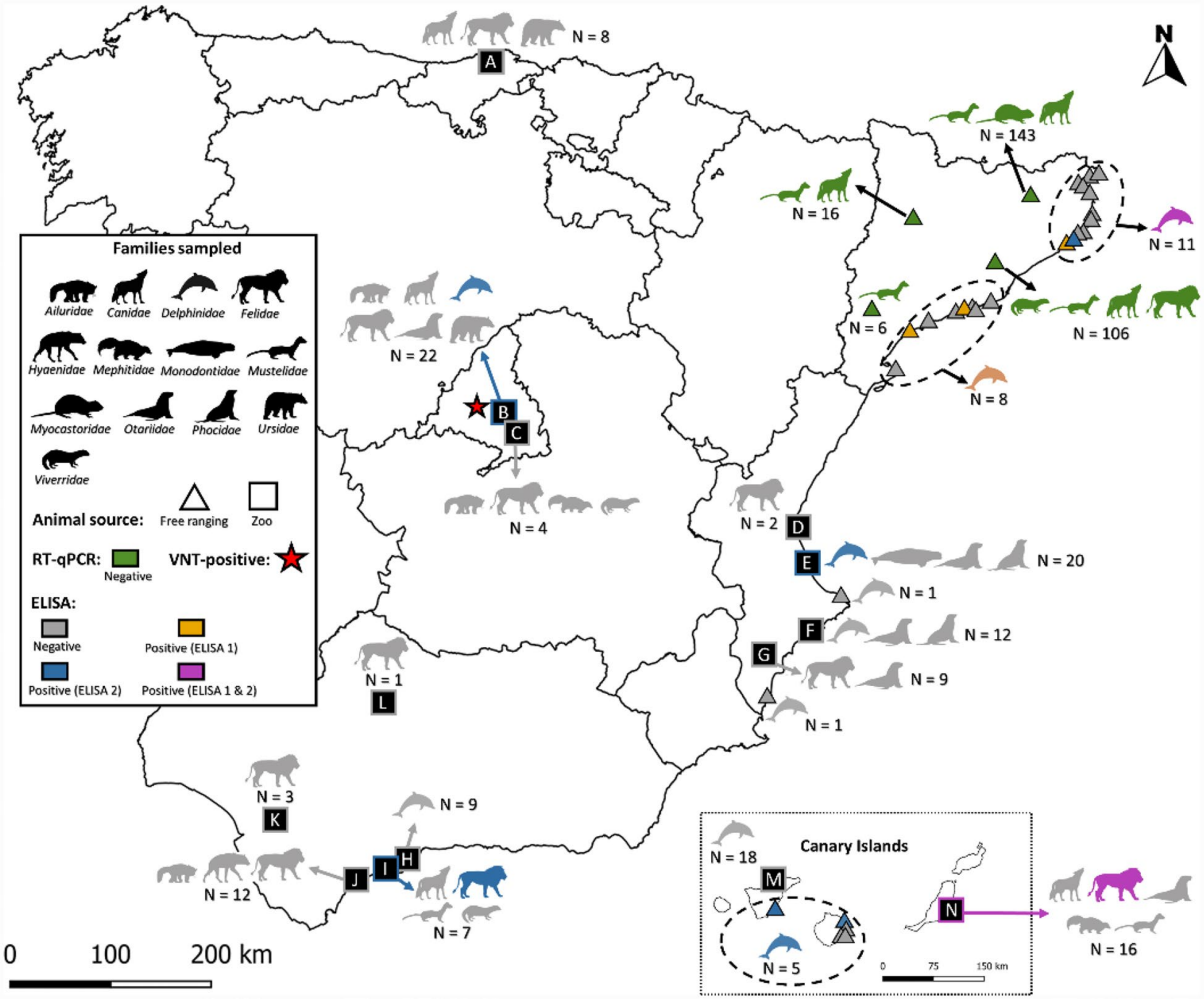
**Geographical distribution of animals sampled in Spain during the COVID-19 pandemic (2020–2023), categorized by family taxonomy.** Zoos and rehabilitation centres are represented by letters (**A**–**N**), and the animal source (free-ranging or zoo) is indicated by the corresponding figure (triangle or square, respectively). Positive results in diagnostic tests are indicated by yellow, blue and violet when positive according to ELISA 1, ELISA 2 or both ELISAs, respectively. ELISA 1 corresponds to the SARS-CoV-2 NeutraLISA assay (EUROIMMUNE, Germany), while ELISA 2 refers to the ID Screen® SARS-CoV-2 Double Antigen Multispecies assay (Idvet, France). VNT positivity is marked with a red star.

Serum samples were obtained from 137 out of 420 animals belonging to 33 different species, encompassing all captive animals and exclusively free-ranging aquatic species from different regions of Spain (Figure 1; Table 1). Serum samples (*n* = 33) collected before the COVID-19 pandemic (prior to 2019; pre-pandemic period) from 17 different species were included and considered negative controls (Additional file 1).

Oropharyngeal swabs (OS), rectal swabs (RS) and lung tissue samples were collected from the remaining animals (283/420). The numbers of free-ranging terrestrial animals sampled in the Catalonia region were 141 American minks, 2 Beech marten (*Martes foina*), 3 common genets (*Genetta genetta*), 48 coypus, 48 Eurasian badgers (*Meles meles*), 25 Eurasian otters (*Lutra lutra*), 1 European wildcat (*Felis silvestris silvestris*), and 15 red foxes (Figure 1; Table 1). Each type of sample was collected from almost all the animals depending on availability (282 from each type of sample). OS and RS were collected using sterile dry swabs or flocked swabs in 2 mL of viral transport media (VTM) (Deltalab, S.L. Catalunya, Spain). Lung tissue samples were placed into cryotubes with 500 μL of Dulbecco’s modified Eagle medium (DMEM) (Lonza, Basel, Switzerland) supplemented with 100 U/mL penicillin, 100 μg/mL streptomycin, and 2 mM glutamine (all from Gibco Life Technologies, Madrid, Spain) and containing single zinc-plated, steel 4.5-mm beads. All the samples were kept at − 20 °C until they were transported properly to the laboratory for further analysis.

Zoological animals were sampled by Zoo Management veterinarian specialists during routine health assessments or surgical interventions. Sera from free-ranging wildlife were sampled by veterinarians from wildlife rehabilitation centres during routine health assessments. OS, RS, and lung tissues were sampled from free-ranging animals from Catalonia (NE-Spain) during necropsies at the Torreferrusa Wildlife Rehabilitation Centre (license number B2300083). All procedures followed the ethical principles of animal research. Sera from free-ranging cetaceans were obtained from individuals stranded on the Atlantic and Mediterranean coasts of Spain, and sampling was performed according to Spanish legislation. Ethical approval by the Institutional Animal Care and Use Committee was not, therefore, deemed necessary. American minks and coypus, subjected to population control programs of the National Government of the *Generalitat de Catalunya*, were sampled during necropsies.

### Detection of antibodies against SARS-CoV-2

**Figure 2:**
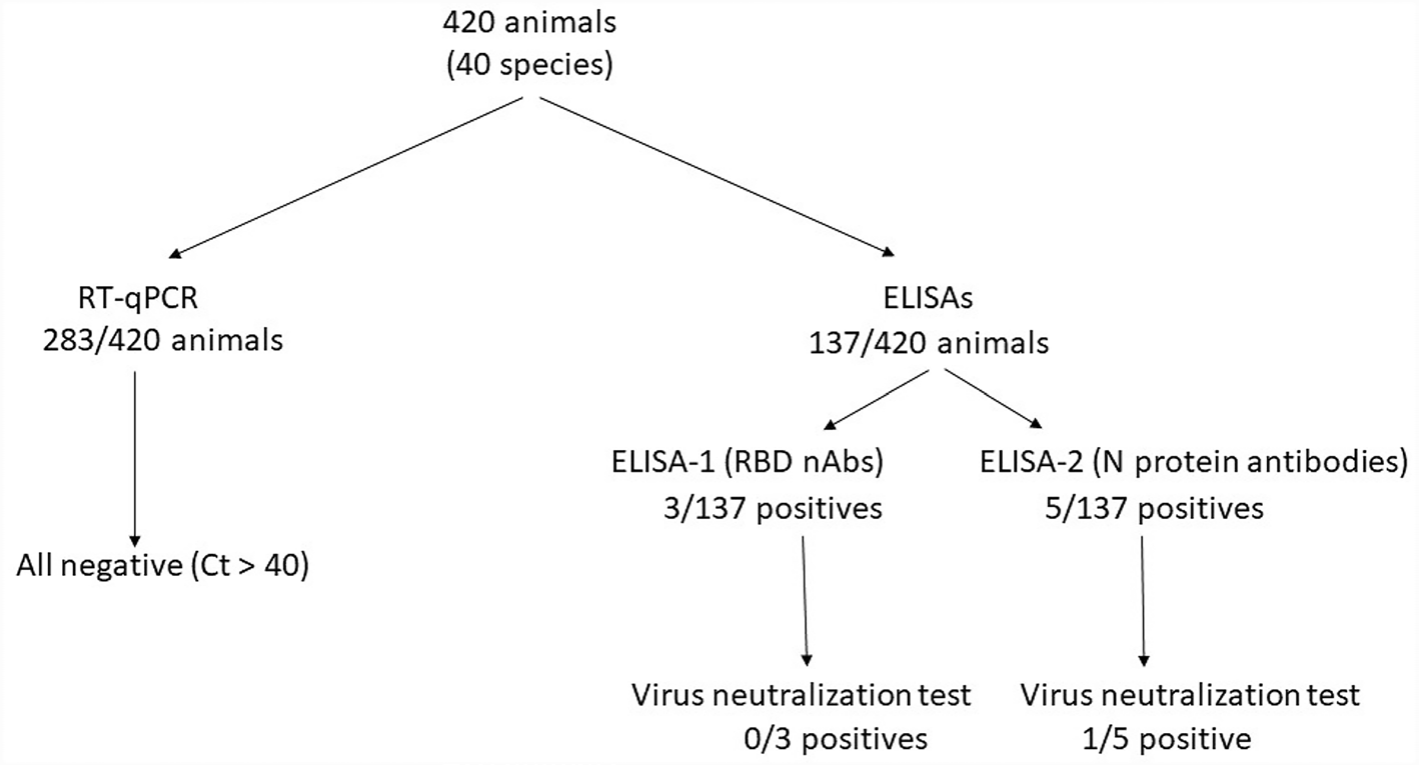
**Number of animals surveyed during the COVID-19 pandemic (2020–2023) and the results of each laboratory technique (RT-qPCR, ELISA-1, ELISA-2, and VNT).** VNT: virus neutralization test.

Available serum samples (137 and 33 from the pandemic and pre-pandemic periods, respectively) were tested with two commercial ELISA kits to detect the presence of specific antibodies against SARS-CoV-2 (Figure 2; Additional file 1): (ELISA 1) the SARS-CoV-2 NeutraLISA assay (EUROIMMUNE, Germany), which detects neutralizing antibodies (nAbs) against the RBD, and (ELISA 2) the ID Screen® SARS-CoV-2 Double Antigen Multispecies assay (Idvet, France), which detects antibodies against the viral nucleocapsid (N) protein. Both tests were performed following the manufacturer’s instructions.

Briefly, sera were analysed by ELISA 1, which provides S1/RBD-coated 96-well plates for capture and soluble biotinylated ACE2 receptor detection. Briefly, each sample was diluted 1:5 with sample dilution buffer containing soluble biotinylated ACE2, and each mixture was added to S1/RBD precoated wells. Following an incubation period at 37 °C for 60 min, three washing steps were conducted with 300 μL of washing solution each. Subsequently, streptavidin-HRP conjugate and the substrate solution were added, and the plate was incubated at RT for 15 min. Finally, the stop solution was added to visualize the optical density (OD) at 450 nm. The results are expressed as an inhibition percentage (% IH) according to the formula provided by the manufacturer’s protocol: % IH = 100% − (sample OD × 100%/mean OD of blank controls). An inhibition (IH) < 20 was considered negative neutralization, an IH of 20–35% was considered doubtful, and an IH ≥ 35% was considered positive neutralization. In addition, the samples were tested in parallel using ELISA 2, which provides N-coated plates for capture and HRP-conjugated SARS-CoV-2 N for detection. In summary, 25 μL of each sample was diluted 1:1 with dilution buffer and added to each well of a 96-well plate. Following incubation at 37 °C for 45 min, five washing steps with 300 μL of each washing solution were performed. Then, 100 μL of recombinant N protein-HRP conjugate was added to each well and incubated at RT for 20 min. Finally, 100 μL of the stop solution was added, and the OD at 450 nm was read. The results were analysed by the following formula provided by the manufacturer’s protocol: Sample/Positive control (S/P) % = [(OD sample − OD negative control)/(OD positive control – OD negative control)] × 100. Samples with S/P% ≤ 50% were considered negative, 50% < S/P% < 60% were considered doubtful, and S/P% ≥ 60% were considered positive. All positive and doubtful samples were tested in duplicate by both ELISAs.

Furthermore, to confirm the presence of nAbs against SARS-CoV-2, positive and doubtful samples from at least one of the ELISAs were further tested in duplicate using a virus neutralization test (VNT) as previously described (Figure 2) [30]. Briefly, serum samples were inactivated (37 °C; 1 h) and diluted 1:10. Then, twofold serial dilutions were performed in supplemented DMEM. The samples were mixed 1:1 with 100 TCID_50_ of an isolate of SARS-CoV-2 (D614G strain) from a COVID-19 patient (GISAID ID EPI ISL 471472) and incubated at 37 °C for 1 h. Subsequently, the mixtures were transferred onto Vero E6 (ATCC® repository, Manassas, VA, USA, CRL-1586™) cell monolayers and cultured for 3 days at 37 °C and 5% CO_2_. Nine ELISA-negative samples from eight different species were randomly selected and included as negative controls for VNT analyses (Additional file 1). The presence of cytopathic effect (CPE) was evaluated at 3 days post-inoculation using the CellTiter-Glo luminescent cell viability assay (Promega, Madison, WI, USA) following the manufacturer’s protocol. Luminescence was measured as relative luminescence units (RLUs) in a Fluroskan Ascent FL luminometer (ThermoFisher Scientific, Waltham, MA, USA). The 50% serum virus neutralization titre (SNT_50_) was defined as the reciprocal dilution of the sample at which 50% of the cells were protected. The dose–response curve of the serum sample was adjusted to a nonlinear fit regression model calculated with a normalized logistic curve with variable slope. Uninfected cells and virus-infected cells were used as negative and positive controls for data normalization, respectively (%Neutralization = (RLUmax − RLUexperimental)/(RLUmax − RLUmin) × 100). All the statistical analyses were performed with GraphPad Prism 8.4.3 (GraphPad Software, Inc., San Diego, CA, USA).

### RNA extraction and detection of SARS-CoV-2 by RT-qPCR

A total of 283 out of 420 animals were tested for acute SARS-CoV-2 infection by the detection of viral RNA in OS, RS, and lung tissue (Table 1; Figure 2).

The dry sterile OS and RS were transferred into cryotubes containing 500 μL of supplemented DMEM and vortexed. The samples obtained by using DeltaSwab Virus with VTM were directly vortexed. Lung tissue was mechanically homogenized at 30 Hz for 1 min using a TissueLyser II (QIAGEN GmbH, Hilden, Germany) and centrifuged for 3 min at 10 000 rpm.

All samples were subjected to viral RNA extraction using the Indimag Pathogen Kit (Indical Biosciences Leipzig, Germany) on a BioSprint 96 workstation (Qiagen, Hilden, Germany) following the manufacturer’s instructions. Subsequently, SARS-CoV-2 RNA was quantified by RT-qPCR using a previously described protocol, which targets the envelope protein (E)-encoding gene [31] with some modifications [30]. The RT-qPCR was performed using AgPath-ID™ One-Step RT-PCR Reagents (Applied Biosystems, Life Technologies, Waltham, MA, USA), and amplification was performed using a 7500 Fast Real-Time PCR System (Applied Biosystems, Life Technologies). Samples with a Cq value < 40 were considered positive for SARS-CoV-2 genomic detection.

## Results

Eight out of the 137 (pandemic) serum samples tested positive for antibodies against SARS-CoV-2 by ELISA (Figures 1 and 2). These samples corresponded to five free-ranging striped dolphins (*Stenella coeruleoalba*), two captive bottlenose dolphins (*Tursiops truncatus*) and one captive Sumatran tiger (*Panthera tigris sumatrae*) (Table 2). Among these ELISA-positive samples, only one captive bottlenose dolphin from Zoo B (Madrid Province) tested positive for VNT, although with low titres of nAbs (SNT_50_ 38.15) (Figure 1; Table 2). Two out of the 33 pre-pandemic samples, one captive Eurasian lynx (*Lynx lynx*) and one free-ranging Risso dolphin (*Grampus griseus*), also tested positive by ELISA but negative by VNT (Figure 1; Table 2).

**Table 2:** **Results of the serological analysis, including ELISAs detecting RBD (ELISA 1) and N protein (ELISA 2) antibodies against SARS-CoV-2 and VNT**

Species	ELISA 1		ELISA 2		VNT	
	IH%	Results	S/P%	Results	SNT_50_	Results
Bottlenose dolphin (*Tursiops truncatus*)	< 20	Negative	186.5	Positive	38.2	Positive
Bottlenose dolphin (*Tursiops truncatus*)	< 20	Negative	712.2	Positive	< 20	Negative
*Striped dolphin* (*Stenella coeruleoalba*)	51.4	Positive	≤ 50	Negative	< 20	Negative
*Striped dolphin* (*Stenella coeruleoalba*)	< 20	Negative	902.2	Positive	< 20	Negative
*Striped dolphin* (*Stenella coeruleoalba*)	< 20	Negative	69.8	Positive	NA	NA
*Striped dolphin* (*Stenella coeruleoalba*)	35.7	Positive	≤ 50	Negative	NA	NA
*Striped dolphin* (*Stenella coeruleoalba*)	49.0	Positive	≤ 50	Negative	< 20	Negative
Sumatran tiger (*Panthera tigris sumatrae*)	< 20	Negative	146.6	Positive	< 20	Negative
*Eurasian Lynx* (*Lynx lynx*)	81.5	Positive	93.0	Positive	< 20	Negative
Risso’s dolphin (*Grampus griseus*)	< 20	Negative	699.1	Positive	< 20	Negative

Only ELISA-positive samples are included. The following serum samples were considered positive for each test: IH% ≥ 35 (ELISA 1), S/P% ≥ 60 (ELISA 2) and SNT_50_ > 20 (VNT).

*NA* not analysed due to insufficient volume.

All animals tested (*n* = 283) for SARS-CoV-2 RNA in OS, RS and/or lung tissue were negative by RT-qPCR (Ct ≥ 40).

## Discussion

Owing to the capacity of SARS-CoV-2 for interspecies transmission, surveillance studies in wildlife are necessary to monitor viral spread and maintenance in wildlife populations, which subsequently may act as reservoirs, promoting genetic evolution and posing a risk for global health. In the present study, we performed an extensive survey of SARS-CoV-2 infection or past exposure in a variety of captive and free-ranging terrestrial and aquatic species of Spain during the whole pandemic period (2020–2023). We detected exposure to SARS-CoV-2 (nAbs) in a captive bottlenose dolphin living in a zoological park, whereas no other animals showed evidence of SARS-CoV-2 exposure or acute infection according to molecular and a set of serological analyses.

The bottlenose dolphin is commonly housed in zoological collections. Consequently, close contact between this species and zookeepers or zoo visitors increases the probability of cross-species transmission of infectious pathogens such as SARS-CoV-2. Importantly, the bottlenose dolphin was predicted to have a high risk of infection with SARS-CoV-2 according to in silico studies due to the high homology between the ACE2 receptor of this host and that of humans [4]. Only five of the 25 critical SARS-CoV-2 S-binding residues differ between the bottlenose dolphin and hACE2 receptors, and there is only one nonconserved amino acid substitution between them [4]. Accordingly, cells expressing ACE2 from bottlenose dolphins allowed cell entry of pseudoviruses expressing the spike (S) protein of an early pandemic isolate of SARS-CoV-2 and Delta and Omicron variants [32]. Additionally, the expression of ACE2 in the respiratory tract of the bottlenose dolphin also supports the potential susceptibility of this animal host under natural conditions [33]. Taken together, the findings of these studies may explain the putative SARS-CoV-2 exposure of the seropositive bottlenose dolphin in the present study. This animal was originally from a zoological park in Madrid and was sampled in May 2020 during the first wave of the COVID-19 pandemic. N protein antibodies against SARS-CoV-2 were detected by ELISA in serum samples, and afterwards, the presence of nAbs was confirmed by VNT. Considering that the majority of nAbs are known to target the RBD and not the N protein of SARS-CoV-2, an ELISA detecting RBD-nAbs could provide false-negative results. This is consistent with the low sensitivity of the commercial kit found in previous comparative analyses of a variety of serological assays using VNT as a reference, although human samples were tested [34]. ELISAs from our study also revealed seropositivity against SARS-CoV-2 in other cetaceans (*Tursiops truncatus* and *Stenella coeruleoalba*) samples, including one pre-pandemic sample, but all tested negative by VNT. Considering that VNT is the gold standard technique for detecting specific nAbs, these results suggest potential cross-reactivity with antibodies against other known or unknown CoVs infecting cetaceans [35].

Cetaceans can be infected with CoVs from the genera *Alphacoronavirus* and *Gammacoronavirus,* including bottlenose dolphin CoVs (BdCoVs) [36]. To date, no cases of SARS-CoV-2 infection have been reported in captive or free-range cetacean animal species; this is the first study detecting SARS-CoV-2 exposure in a captive dolphin. Audino et al. described the absence of SARS-CoV-2 infection in a variety of marine mammals from the Italian coastline, consistent with negative results obtained by RT-qPCR and/or immunohistochemistry (IHC) tests. Nevertheless, past infection or exposure in those animals tested could not be completely ruled out since both RT-qPCR and IHC detect only acute infections, contrary to serological analyses [33]. Due to the likely susceptibility of dolphins to SARS-CoV-2, future studies should focus on elucidating the potential impact of this virus on dolphin individual and population health [33].

The SARS-CoV-2 genome has been detected in wastewater and rivers and is used even for epidemiological and predictive studies of the incidence of SARS-CoV-2 in human populations [37, 38]. This fact suggests the possibility of exposure to SARS-CoV-2 in aquatic and semiaquatic animals and, thus, supports the relevance of monitoring this group of animals. Indeed, there is a report describing SARS-CoV-2 positivity in water pool samples from a zoological park in Belgium in which two infected hippos (*Hippopotamus amphibious*) were living [12]. However, it should be noted that water treatment procedures (wastewater or pools) and marine water factors (salinity, pH or dilution effect) may contribute to SARS-CoV-2 inactivation and reduce the viral load, decreasing the risk of infection [33].

In our study, we also included samples from wild mustelid species that live in aquatic environments. These species were predicted to have a low risk of infection by in silico studies due to the low binding affinity between the SARS-CoV-2 RBD and the host ACE2 receptor [4]. However, in vivo experiments and previous reports describing natural infections have already demonstrated their high susceptibility to SARS-CoV-2, probably due to the high levels of ACE2 in the respiratory tract [39–42]. SARS-CoV-2-seropositivity or infection in mustelids has been reported mainly in the livestock industry (American minks), households (ferrets; *Mustela putorius furo*) and zoos (Asian small-clawed otter) but also in free-ranging environments (Eurasian otters, American mink, pine martens [*Martes martes*] and badgers) [3]. To date, most of the studies have focused on monitoring infection in domestic rather than in free-ranging mustelids, probably due to the difficulties involved in sampling them. Notably, our study prioritized the surveillance of free-ranging mustelid species, with a particular emphasis on the American mink. None of the sampled animals tested positive for acute infection. It is important to note that viral exposure cannot be excluded in this species since serum samples for the detection of SARS-CoV-2 antibodies were unavailable, as sampling was conducted post-mortem. Experimental infections have demonstrated that American minks usually manifest severe COVID-19-like conditions, including pronounced lesions in both the nasal mucosa and lungs, similar to those observed in severe cases in humans [39, 43]. In natural infections, minks succumb to mortality mainly due to interstitial pneumonia associated with viral infection [44]. Consequently, detecting PCR-positive minks for SARS-CoV-2 infection may pose challenges, given their high susceptibility and mortality rates, unless diseased individuals are sampled or during active outbreak investigations.

The present study did not detect SARS-CoV-2 RNA or SARS-CoV-2 antibodies in any other captive or free-ranging wild animals. In contrast to our results, many natural infections have been described in wild mammals, mainly carnivore species, and most of them occurred in zoological parks [3, 9]. Zoos are suitable for viral cross-species transmission due to the large diversity of animal species and frequent human–animal interactions. In particular, medium- and large-sized wild felids have shown high susceptibility to SARS-CoV-2 infection even when they present no to mild-moderate clinical signs (respiratory and digestive) [45–48]. Notably, the Delta (B.1.612.2) variant has been suggested to cause more severe disease in this group of species and is considered a contributing cause of death in some animals [49]. Additionally, feline species can generate a significant humoral immune response against SARS-CoV-2 after natural infection via the presence of RBD nAbs and limited levels of antibodies against the N protein [45, 50, 51]. RBD nAbs persist for at least 4 months, and total nAbs may be present at least 18 months after natural infection in lions [45, 52]. In our study, one Sumatran tiger exhibited positive results for N protein antibodies, and one pre-pandemic Eurasian lynx tested positive for RBD nAbs and N protein antibodies. Nevertheless, the VNT results suggested false-positive results in both cases and potential cross-reactivity of antibodies from other feline CoVs [53]. A similar study conducted in zoo animals from France reported positive ELISA results for N protein antibodies and RBD nAbs in serum samples from three Springbok sheep (*Antidorcas marsupialis*), three Cameroon sheep (*Ovis aries Cameroon*) and two vicunas (*Vicugna vicugna*) [54]). However, these results were not confirmed by VNT, leading us to consider them potential false positives (51). Regarding free-ranging felid animals, one study described the case of an infected leopard (*Panthera pardus fusca*) by the Delta (B.1.617.2) variant in India, and a recent study demonstrated the case of virus exposure in free-ranging Iberian lynx (*Lynx pardinus*) in southern Spain with high titres of nAbs [55].

Notably, the animals included in this study were tested for both acute infection and/or exposure to SARS-CoV-2. Thus, the number of samples for some species was low and sporadic over time, which could have contributed to the failure to detect positive animals. Additionally, the animals could have overcome the infection at the time of sampling, or their immune responses could have decreased below the limit of detection of the techniques. Importantly, ELISAs cannot have the same levels of sensitivity or specificity when used in wild species as in domestic species or humans [56]. The difficulty in obtaining species-specific positive and negative controls for serological analyses hinders the validation of these diagnostic tests in wildlife. Additionally, it would be necessary to include other groups of species (e.g., bats and ungulates), so we could acknowledge whether some other species could be infected and missed by our study. Monitoring wildlife species for emerging diseases poses many challenges. Wildlife that runs freely in the wild can be vast and dispersed, making it difficult to access and sample individuals effectively. Additionally, monitoring wildlife animals requires specialized techniques, trained professionals, special equipment or permissions to capture and handle specific species [56]. Overall, limitations and challenges to wildlife disease surveillance stand out.

The results from our study provide a favourable perspective regarding the absence of SARS-CoV-2 in wild animals from captive and free-range environments in Spain. However, the promiscuity of SARS-CoV-2 for multiple animal species and its ability to cross-species barriers reinforce the importance of continuing to monitor wildlife. In particular, surveillance of SARS-CoV-2 infection in species living at high densities, in potential animal reservoirs and in areas with close animal-human interactions has been performed. Our study agrees with previous in silico and in vitro studies showing that SARS-CoV-2 infection in marine mammals is feasible. This finding also supports preventive biosecurity measures when interacting with cetaceans and other potentially susceptible species in cases of suspected or confirmed COVID-19.

### Supplementary Information

**Additional file 1. Serum samples were collected during the pre-pandemic period (prior to 2019) and were used as negative controls for ELISA (*n* = 33) and VNT (*n* = 9).**
