# Supplementary material for: Survey of severe acute respiratory syndrome coronavirus 2 in captive and free-ranging wildlife from Spain
Source: Vet Res. 2024 Jul 19;55:90. doi: 10.1186/s13567-024-01348-0 (PMC11264983; doi:10.1186/s13567-024-01348-0)
Supplement: Supplementary file 1 — Additional file 1. Serum samples were collected during the pre-pandemic period (prior to 2019) and were used as negative controls for ELISA (n = 33) and VNT (n = 9). [file 13567_2024_1348_MOESM1_ESM.docx]

**Additional file 1. Serum samples collected during the pre-pandemic period (prior to 2019) and considered as negative control for ELISA (*n* = 33) and VNT (*n* = 9).**

| Negative control | Species | Family | Number of animals |
| --- | --- | --- | --- |
| ELISA | Red fox (*Vulpes vulpes*) | *Canidae* | 1 |
|  | Bottlenose dolphin (*Tursiops truncatus*) | *Delphinidae* | 4 |
|  | Common dolphin (*Delphinus delphis*) | *Delphinidae* | 1 |
|  | Risso’s dolphin (*Grampus griseus*) | *Delphinidae* | 3 |
|  | Striped dolphin (*Stenella coeruleoalba*) | *Delphinidae* | 5 |
|  | African lion (*Panthera leo*) | *Felidae* | 1 |
|  | Asian tiger (*Panthera tigris tigris*) | *Felidae* | 2 |
|  | Bobcat (*Lynx rufus*) | *Felidae* | 1 |
|  | Cheetah (*Acinonyx jubatus*) | *Felidae* | 2 |
|  | Eurasian lynx (Lynx lynx) | *Felidae* | 3 |
|  | Katanga lion (*Panthera leo bleyenberghi*) | *Felidae* | 2 |
|  | Ocelot (*Leopardus pardalis*) | *Felidae* | 1 |
|  | Sri Lankan leopard (*Panthera pardus kotiya*) | *Felidae* | 1 |
|  | Sumatran tiger (*Panthera tigris sumatrae*) | *Felidae* | 1 |
| Total | **Negative control ELISA** |  | **33** |
| VNT | Risso’s dolphin (*Grampus griseus*) | *Delphinidae* | 2 |
|  | Striped dolphin (*Stenella coeruleoalba*) | *Delphinidae* | 1 |
|  | African lion (*Panthera leo)* | *Felidae* | 1 |
|  | Asian tiger (*Panthera tigris tigris*) | *Felidae* | 1 |
|  | Cheetah (*Acinonyx jubatus*) | *Felidae* | 1 |
|  | California sea lion (*Zalophus californianus*) | *Otariidae* | 1 |
|  | South American sea lion (*Otaria flavescens*) | *Otariidae* | 1 |
|  | Asian black bear (*Ursus thibetanus*) | *Ursidae* | 1 |
| Total | **Negative control VNT** |  | **9** |
